# Evaluation of mid-infrared milk spectroscopy as a tool for genetic improvement of nitrogen utilization in dairy cow

**DOI:** 10.3168/jdsc.2025-0974

**Published:** 2026-03-27

**Authors:** E. Tavernier, M. Frizzarin, D.P. Berry

**Affiliations:** 1 IDELE, 49071 Beaucouzé, France; 2 Agroscope, 1725 Posieux, Switzerland; 3 Teagasc, Animal & Grassland Research and Innovation Centre, Moorepark, Fermoy P61 P302, Co. Cork, Ireland

## Abstract

Nitrogen losses from dairy systems, such as nitrate leaching and nitrous oxide emissions, can disturb the nitrogen cycle. An effective mitigation strategy involves selecting cows with improved nitrogen use efficiency and reduced nitrogen excretion. Accurate genetic evaluations are dependent on routine access to large quantities of individual cow phenotypic data on the trait of interest, a feat that is not simple for nitrogen utilization, especially in grazing dairy cows. A workaround is to incorporate information from a proxy trait into a multi-trait selection index; for example, predictions from mid-infrared (MIR) spectroscopy of milk, a widely used method globally for predicting major milk components. This study used nitrogen utilization data and accompanying milk mid-infrared spectra from 1,186 Irish grazing cows across 4 experimental farms between the years 2007 and 2018; the 2 nitrogen utilization metrics explored were nitrogen use efficiency (NUE) and nitrogen balance (NBAL). The accuracy of (indirect) genetic selection for nitrogen utilization using milk MIR spectra-based predictions of nitrogen utilization traits was quantified; neural networks or partial least squares regression were used in the phenotypic prediction. The MIR predictions used were those derived from cow-level cross-validation or leave-one-farm-out methods. Irrespective of prediction method or validation approach, the partial phenotypic correlations between the observed nitrogen utilization values and their respective predictions were stronger for NUE (0.46–0.51) than for NBAL (0.23–0.28). Based on cow-level cross-validation, the genetic correlations between the observed nitrogen utilization values and those predicted from the milk MIR ranged from 0.69 (SE = 0.06) to 0.84 (SE = 0.06) for NUE and from 0.57 (0.07) to 0.71 (SE = 0.10) for NBAL. Using leave-one-farm-out validation, the genetic correlations between the observed nitrogen utilization values and those predicted from the milk MIR ranged from 0.64 (SE = 0.07) to 0.75 (SE = 0.06) for NUE and from 0.43 (SE = 0.08) to 0.73 (SE = 0.09) for NBAL. Thus, even though the accuracy of phenotypic prediction is low, milk mid-infrared spectral predictions serve as a reliable proxy for genetic selection aimed at improving nitrogen utilization.

## Highlights

• The accuracy of phenotypic predictions for nitrogen-utilization metrics was low.
• Strong genetic link between observed and prediction nitrogen efficiency.
• Prediction from milk spectra can aid genetic progress on nitrogen utilization.

## Graphical Abstract

An effective mitigation strategy against nitrogen loss from dairy farms involves genetically selecting cows with improved nitrogen-use efficiency and reduced nitrogen excretion. Such a strategy requires routine access to large quantities of individual cow data. A promising approach to generate these data is the use of mid-infrared spectroscopy of milk to estimate how well cows utilize nitrogen. Predictions from the milk infrared information were genetically correlated with actual nitrogen-use efficiency and excretion levels. This, therefore, suggests that such predictions from routinely available milk mid-infrared spectra data could assist in genetically selecting more nitrogen-efficient grazing dairy cows with a lower nitrogen footprint. Error bars in image represent standard error.

**Figure:**
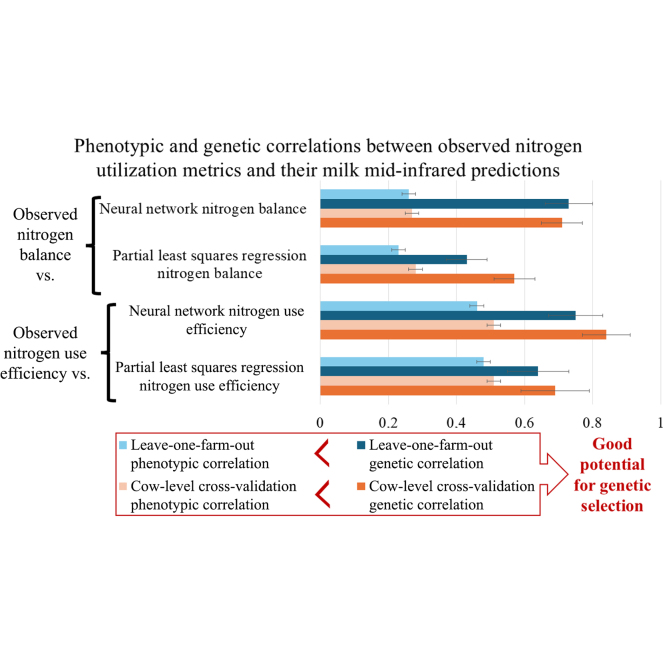

Measuring nitrogen utilization in grazing dairy cows is challenging. This is particularly problematic for breeding initiatives attempting to improve nitrogen utilization, as an efficient breeding program ideally requires routine access to individual cow data for the trait or traits of interest. Key nitrogen utilization traits include nitrogen use efficiency (**NUE**; Lopez-Villalobos et al., 2018; Grelet et al., 2020; Aizimu et al., 2021) and nitrogen balance (**NBAL**; Zamani et al., 2011; Aarons et al., 2017; Grelet et al., 2020). Nitrogen use efficiency is defined as the nitrogen output going into products (e.g., milk, meat) divided by nitrogen available. It represents the share of nitrogen intake that is used for production. By improving NUE, more products can be generated from the same amount of resources. Nitrogen balance, defined as the difference between nitrogen intake and nitrogen output, serves as an indicator of nitrogen excretion. A reduction in NBAL corresponds to a lower potential nitrogen footprint in dairy cows and mitigates negative environmental impacts, such as nitrogen leaching. Both metrics rely on accurate estimates of nitrogen intake for individual cows. Generating individual cow feed intake records in grazing systems is resource-intensive, thereby limiting the number of direct phenotypes that can be recorded, especially on commercial farms; this limits the accuracy of genetic selection. Recent studies have explored mid-infrared (**MIR**) spectroscopy of milk as a means of predicting nitrogen utilization traits in dairy cows (Grelet et al., 2020; Shi et al., 2023; Frizzarin et al., 2024). Although the documented accuracy of these MIR-based phenotypic predictions vary, only Frizzarin et al. (2024) used data from grazing dairy cows and reported poor predictive ability of both NUE and NBAL when the calibration data used to generate the predictions originated from herds different to those in which they were validated. Although MIR spectroscopy has limitations in directly predicting phenotypic nitrogen utilization of individual dairy cows, it may still be useful in genetic evaluations. This is especially true when no residual correlation between the traits exists, or when the residual correlation is opposite in direction to the genetic correlation, or, in fact, if the traits have low heritability. The objective of the present study was to evaluate the usefulness of milk MIR phenotypic predictions of nitrogen utilization in dairy cows to contribute to delivering accurate genetic evaluations for nitrogen utilization, specifically identifying cows that excrete less nitrogen and utilize more of the nitrogen they ingest.

The phenotypic data available from this population has already been described in detail by Tavernier et al. (2023). In summary, data were collected across 4 Teagasc experimental farms in Southern Ireland between the years 2007 and 2018. Individual cow grass and concentrate DMI, along with associated information on their respective CP and energy, were available. Also recorded per cow were daily milk yield, weekly milk composition, bimonthly (twice a month) liveweight, and weekly individual consecutive evening (p.m.) and morning (a.m.) milk spectra. All milk samples were analyzed using the same MIR spectrometer (MilkoScan FT6000, Foss Electric A/S, Hillerød, Denmark), generating 1,060 transmittance spectral values, which were converted to absorbance. Spectra were edited using the approach described by Frizzarin et al. (2024), which included the removal of high-noise-level regions from each spectrum, as proposed by Visentin et al. (2015); 531 wavelengths remained.

For each cow in the dataset, the fraction of Holstein-Friesian (**HF**) and Jersey (**JE**) was available; the other breed fractions were summed into a single group, Others. The heterosis coefficient was computed as $1-\;\sum_{i=1}^{n}\mathrm{sir}e_{i}\cdot dam_{i},$ and the recombination loss coefficient as $1-\;\sum_{i=1}^{n}\frac{{\mathrm{sire}}_{i}^{2}+{\mathrm{dam}}_{i}^{2}}{2},$ where sire*_i_* and dam*_i_* were the proportions of breed *i* in the sire and dam, respectively. The heterosis coefficient (in percentage units) was categorized into 10 classes: [0,10], [10,20], [20,30], [30,40], [40,50], [50,60], [60,70], [70,80], [80,90], and [90,100]. The recombination loss coefficient was divided into 9 classes: [0,10], [10,20], [20,30], [30,40], [40,50], [50,60], [60,70], [70,80], and [80,100].

Two additional cow-level features were generated for later use in the calculation of nitrogen utilization: daily liveweight change and daily energy balance. Daily liveweight was modeled from the bimonthly liveweight records using a mixed model as described by Tavernier et al. (2023) for this dataset; the output was predicted liveweight for each DIM, which were then used to calculate liveweight change. Energy balance per animal was calculated to determine how much nitrogen was being stored or mobilized. If the calculated energy balance was positive, part of the energy and nitrogen ingested was deemed to be stored in reserve. In contrast, negative energy balance suggested that energy and nitrogen reserves were being mobilized to support bodily functions. Energy balance was determined by deducting the energy used for milk production, pregnancy, growth, and maintenance from the total energy intake (Tavernier et al., 2023). The sources and sinks of energy were calculated using the methods outlined by Sauvant et al. (2018), as reported in Tavernier et al. (2023) when applied to the data used in the present study.

Nitrogen available (**N_avail_**) and nitrogen output into product (**N_out_**) were computed as detailed by Tavernier et al. (2023). In summary, N_avail_ was the sum of the nitrogen intake (i.e., sum of grass and concentrate DMI multiplied by their respective CP content and converted into nitrogen equivalents) and nitrogen available from body tissue catabolism. Nitrogen was deemed available in a period of negative energy balance and was computed as the energy balance times 33 g of protein per unités fourrage laitière (unit of the energy balance; Sauvant et al., 2018) divided by 6.25 (Jones, 1931). Nitrogen output was calculated as the sum of the nitrogen used for milk, conceptus, and growth, as well as stored in reserve. The nitrogen in milk was the sum of milk urea nitrogen and the true protein converted into nitrogen equivalents. The nitrogen used for the conceptus was 4/3 times the nitrogen used for the growth of the fetus, as computed by Agabriel and de la Torre (2018). The nitrogen used for growth of the cow was calculated as the difference in the modeled cow liveweight between the day of the milk recording and the previous day. Finally, the quantity of nitrogen stored when the cow was in positive energy balance was calculated as 33/6.25 times the energy balance.

Two daily nitrogen utilization metrics were defined. Nitrogen use efficiency was defined as the nitrogen output divided by the nitrogen available: NUE = N_out_/N_avail_. Nitrogen balance was defined as the nitrogen available less the nitrogen output: NBAL = N_avail_ − N_out_.

Moreover, both nitrogen utilization metrics were predicted from the milk MIR using the approaches described by Frizzarin et al. (2024) for this dataset. The MIR-based prediction model used both morning and evening milk spectra along with daily milk yield; both partial least squares regression (**PLSR**) and neural network (**NN**) were explored as the method for prediction. To evaluate model performance, 2 validation scenarios were applied: cow-level cross-validation, which is common practice, and leave-one-farm-out cross-validation, which accounts for differences in environmental and farm features and mimics prediction for farms not included in model training, reflecting scenarios encountered during deployment (Yilmaz Adkinson et al., 2024). For the cow-level cross-validation scenario, cows were divided into 4 folds at random, and the nitrogen utilization metrics of cows in 1 fold were predicted from those in the 3 other folds; no cow appeared in both the calibration and the validation datasets. For the leave-one-farm-out validation scenario, the nitrogen utilization metrics of the cows in 1 farm were predicted based on a calibration (i.e., nitrogen utilization metrics and milk MIR) dataset of the cows in the other 3 farms. The final dataset comprised 3,497 individual cow nitrogen utilization records from 2,019 lactations on 1,186 Irish grazing dairy cows; all had actual and MIR-predicted values for both NUE and NBAL.

Variance components for the observed and MIR-predicted NUE and NBAL phenotypes were estimated in ASReml (Gilmour et al., 2009) using a linear mixed model:

[1] Yijlmno=μ+parj·∑k=13bj,kDIMk+Hetl+Recm+JE+Others+CGn+ai+pe_withini,o+pe_acrossi+eijlmno,

where Y*_ijlmno_* was the trait of interest; *µ* was the intercept; $\mathrm{pa}r_{j}\cdot \sum_{k=1}^{3}b_{j,k}\mathrm{DI}M^{k}$ was the third-order Legendre polynomial regression on DIM in a 2-way interaction with parity, with the coefficients b*_j_*_,_*_k_* being the fixed regression effects of the *k*th-order polynomial for the *j*th parity; Het*_l_* was the fixed effect of the heterosis class *l* (*l* = 1–10), and Rec*_m_* was the fixed effect of the recombination loss *m* (*m* = 1–9); JE and Others were the known breed proportion covariates for JE and Others, respectively, for animal *i* (because the known breed proportions of HF, JE, and Others are linearly dependent because their sum equals 1, the breed proportion for HF was not included in the model); CG*_n_* was the fixed effect of the contemporary group *n* (defined as combination of the experimental treatment and the date of measurement); a*_i_* was the random regression coefficient of the animal additive effect, where $a_{i}\left(0,A\sigma_{a}^{2}\right)$ with $\sigma_{a}^{2}$ the direct genetic variance and **A** the numerator relationship matrix without ancestor groups; pe_within*_i_*_,_*_o_* was the random permanent environmental effect within parity *o* (*o* = 1–7) of animal *i*, where $\mathrm{pe}\_\mathrm{withi}n_{i,o}∼\mathrm{iid}N\left(0,\sigma_{w}^{2}\right),$ with $\sigma_{w}^{2}$ denoting the permanent environmental variance within parity *p*; pe_across*_i_* was the random effect of permanent environmental effect across the parities of animal *i*, where $\mathrm{pe}\_\mathrm{acros}s_{i}∼\mathrm{iid}N\left(0,\sigma_{ac}^{2}\right),$ with $\sigma_{ac}^{2}$ representing the permanent environmental variance across parities; the residual term e*_ijlmno_*, where $e_{ijlmno}∼\mathrm{iid}N\left(0,\sigma_{e}^{2}\right),$ with $\sigma_{e}^{2}$ representing the residual variance. Genetic, permanent environmental, and residual covariances between the observed nitrogen utilization metrics and their MIR predictions, as well as between both MIR predictions (i.e., PLSR vs. NN), were estimated from a series of bivariate analyses using the statistical model described; to improve convergence, the permanent environmental variance across parities was not considered in the bivariate analyses.

Heritability estimates and associated genetic SD for the observed and predicted nitrogen utilization traits are shown in Table 1. Both observed and predicted NUE and NBAL had low heritability (0.08–0.26). The genetic SD for NUE ranged from 0.0061 to 0.0093, whereas the genetic SD for NBAL ranged from 16.9 to 21.5 g of nitrogen per day. Moreover, the genetic SD of the nitrogen utilization traits predicted by NN was smaller than that of both the observed and the PLSR-predicted nitrogen utilization metrics. The low heritability for both traits further highlights the necessity of exploring proxy measures considering the practical challenges of collecting gold standard measures on a sufficiently large population to achieve a high accuracy of selection.

**Table 1:** Heritability (h^2^; SE in parentheses) and genetic SD (σ_g_) for both observed and predicted nitrogen use efficiency (NUE) and nitrogen balance (NBAL), as estimated using partial least squares regression or neural network

Trait	Observed		Validation scenario	Partial least squares		Neural networks	
	h^2^ (SE)	σ_g_		h^2^ (SE)	σ_g_	h^2^ (SE)	σ_g_
NUE	0.15 (0.05)	0.0093	Cross-validation	0.22 (0.06)	0.0079	0.09 (0.05)	0.0061
			Leave-one-farm-out	0.23 (0.06)	0.0088	0.08 (0.05)	0.0074
NBAL (g/d)	0.15 (0.06)	21.5	Cross-validation	0.26 (0.06)	20.1	0.14 (0.05)	16.9
			Leave-one-farm-out	0.24 (0.06)	20.0	0.14 (0.05)	17.3

The phenotypic and genetic correlations between the observed and predicted nitrogen utilization metrics are shown in Table 2; the phenotypic and genetic correlations between the observed and predicted nitrogen utilization metrics were similar for both validation approaches. Irrespective of whether predicted using PLSR or NN, the phenotypic correlations between the observed and predicted values were weaker for NBAL than for NUE. Weaker predictions (as represented by correlations) for NBAL than NUE in dairy cows when predicted from milk MIR have been reported in other dairy cow populations (Grelet et al., 2020; Shi et al., 2023). Moreover, weaker correlations between observed and MIR-predicted traits in leave-one-farm-out versus cross-validation were reported in the present study. This trend has also been documented in dairy cows for milk MIR-based predicted methane emissions (Wang and Bovenhuis, 2019) and for milk MIR-based predicted NBAL (Shi et al., 2023). The raw correlations between the observed and predicted NUE from cross-validation and leave-one-farm-out methods were 0.78 and 0.40 for PLSR, and 0.86 and 0.30 for NN, whereas the respective correlations for NBAL were 0.60 and 0.06 for PLSR, and 0.74 and 0.13 for NN. These correlations are the same as those reported by Frizzarin et al. (2024) for the same dataset as the present study, with the exception of 9 fewer cows in the present study.

**Table 2:** Phenotypic and genetic correlations (SE in parentheses) between the observed and predicted nitrogen use efficiency (NUE) and nitrogen balance (NBAL) as estimated using partial least squares regression (PLSR) or neural network (NN); also included are the correlations between the PLSR and NN predictions

Trait	Validation scheme	Correlation	Observed vs. PLSR	Observed vs. NN	NN vs. PLSR
NUE	Cross-validation	Phenotypic	0.51 (0.02)	0.51 (0.02)	0.70 (0.01)
		Genetic	0.69 (0.06)	0.84 (0.06)	0.85 (0.04)
	Leave-one-farm-out	Phenotypic	0.48 (0.02)	0.46 (0.02)	0.67 (0.01)
		Genetic	0.64 (0.07)	0.75 (0.08)	0.81 (0.04)
NBAL	Cross-validation	Phenotypic	0.28 (0.02)	0.27 (0.02)	0.58 (0.02)
		Genetic	0.57 (0.07)	0.71 (0.10)	0.74 (0.06)
	Leave-one-farm-out	Phenotypic	0.23 (0.02)	0.26 (0.02)	0.57 (0.02)
		Genetic	0.43 (0.08)	0.73 (0.09)	0.69 (0.06)

The genetic correlations between the observed and predicted nitrogen utilization metrics were stronger than the phenotypic correlation, with the former ranging from 0.43 to 0.84 across the 2 nitrogen utilization metrics, the 2 statistical methods of prediction, and the 2 validation scenarios. The genetic correlation reflects the strength of the linear relationship between 2 traits attributable to coinherited genomic variants affecting both traits; the correlation could be a manifestation of linkage between causal genomic mutations or pleiotropy. Phenotypic correlations, in contrast, are a function of both environmental and genetic covariances. The phenotypic correlation between traits X and Y was expressed by Falconer and Mackay (1996) as $r_{A}\sqrt{h_{X}^{2}h_{Y}^{2}}+r_{R}\sqrt{\left(1-h_{X}^{2}\right)\left(1-h_{Y}^{2}\right)},$ where r_A_ is the additive genetic correlation between both traits, r_R_ is the residual correlation between both traits, and $h_{*}^{2}$ is the heritability of trait *. Using this equation, the expected phenotypic correlation between the cross-validation PLSR NUE and the observed NUE was 0.49, which is very close to the 0.51 estimated directly in this study (Table 2). Thus, the weaker phenotypic correlations between observed and predicted nitrogen utilization traits, relative to the corresponding genetic correlations, can be explained by the low estimated heritability of both nitrogen utilization metrics, as well as to the estimated weak residual correlations between observed and predicted nitrogen utilization traits (0.15–0.45). Moreover, the residual correlations between observed and predicted NBAL (0.15–0.16) were weaker than those between the observed and predicted NUE (0.43–0.45), contributing to a weaker phenotypic correlation for NBAL than for NUE.

The phenotypic correlations between the predictions for the same trait obtained from the 2 methods explored varied from 0.57 to 0.70, whereas the respective genetic correlations varied from 0.69 to 0.85. This signifies that, although both modeling approaches captured a common genetic basis for the traits, differences in prediction structure or feature weighting resulted in some divergence in model predictions. Nonetheless, the relatively strong correlations between both methods' predictions suggest that nonlinear modeling did not substantially alter the ability to detect the core genetic basis of the traits, although it may have refined the capture of complex interaction effects. Nonetheless, the generally lower heritability and genetic variance estimates for the NN predictions relative to the PLSR predictions suggest that the NN may have smoothed over some of the additive genetic variability while capturing a larger proportion of non-additive or environmental variance. In contrast, the PLSR approach appears to have retained more of the additive genetic component, which could, in part, be due to its linear structure modeling a more direct relationship between the spectra and both nitrogen utilization traits.

In summary, although the phenotypic correlations between the observed and predicted nitrogen utilization metrics were weak, the corresponding moderate to strong genetic correlations estimated in the present study suggest that MIR predictions of nitrogen utilization could potentially be useful proxies for genetically selecting grazing dairy cows with higher NUE and lower NBAL. As true nitrogen utilization phenotypes are challenging to measure, indirect selection may be more effective. Further exploration is required in independent datasets to conclude the best prediction algorithm to use.
